# A lockdown index to assess the economic impact of the coronavirus

**DOI:** 10.1186/s41937-020-00056-8

**Published:** 2020-08-28

**Authors:** Marius Faber, Andrea Ghisletta, Kurt Schmidheiny

**Affiliations:** Universität Basel, Peter Merian-Weg 6, Basel, CH-4002 Switzerland

**Keywords:** Coronavirus, Lockdown, Employment, Short-time work, E24, J21, J23

## Abstract

Like most countries, the Swiss government adopted drastic measures to stop the spread of the coronavirus. These measures were aimed at avoiding close physical proximity between people. The adverse economic consequences of this lockdown policy became immediately apparent, with almost two million workers, or more than every third worker in Switzerland, being put on short-time work within only 6 weeks after the policy’s implementation. In an attempt to promptly assess the heterogeneous consequences of this lockdown policy, we computed a *lockdown index*. This index is based on an occupation’s dependence on physical proximity to other people and corrected for certain essential sectors being exempt from this policy. We find that on average, 31% of jobs in Switzerland have been potentially restricted by the lockdown policy. This average masks considerable heterogeneity along many dimensions, with the strongest effects for the large industries hospitality, construction, and arts and entertainment. With respect to the regional variation, we find the strongest effects for the cantons of Obwalden, Uri, Appenzell Innerrhoden, and Valais. Moreover, low- and middle-income individuals are considerably more restricted than high-income ones. We do not find meaningful differences between men and women or urban and rural areas. Finally, we test the explanatory power of the lockdown index for short-time work and unemployment increases by canton and industry until the end of April 2020 and find that it can explain up to 58% of these short-term employment outcomes.

## Introduction

The fast spread of the coronavirus has caused economic crises all around the globe. In Switzerland, the first patient with COVID-19 was detected on February 25, 2020. The 70 year-old man from the canton Ticino had visited the heavily affected North of Italy before. In the following weeks, the virus quickly spread around the country, such that in the early stages of the pandemic, Switzerland was among the countries with the highest prevalence of positive COVID-19 cases in Europe^1^.

Despite the comparatively high prevalence of positive cases in the first half of March, Switzerland initially adopted only relatively mild measures, such as contact tracing via phone calls or the prohibition of events with more than 1000 participants, to contain the spread of the virus. On March 13, however, the Swiss government started to take more drastic steps and decided to close down all educational institutions as well as to prohibit gatherings with more than 100 people. Only a few days later, on March 16, the government announced the so-called “extraordinary situation,” which entailed even stricter measures. These measures included the closures of shops, restaurants and bars, entertainment and leisure facilities, as well as the prohibition of all public and private events. In addition, the government called on the public to avoid all unnecessary contact, keep distance from others, and to follow the recommended hygiene measures. A few essential industries, such as food stores, the health care sector, and the repair of transport vehicles were explicitly exempt from these measures^2^.

**Fig. 1:**
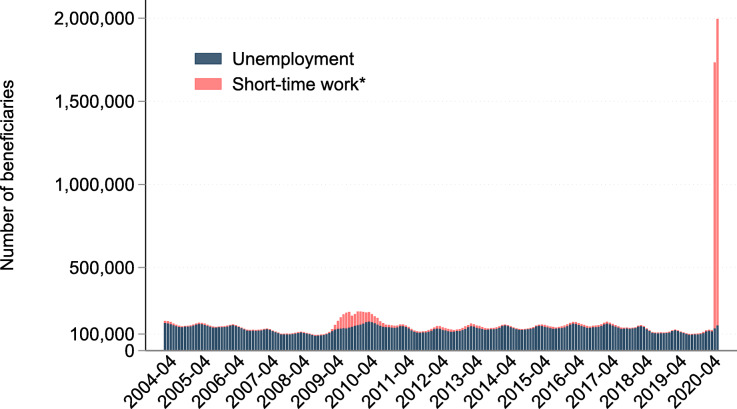
Development of unemployment and short-time work in Switzerland, 2004–2020

This lockdown policy was quickly followed by a sharp rise in the numbers of people registered for short-time work (“Kurzarbeit” in German, “furlough pay” in the UK) and those filing for unemployment insurance as shown in Fig. 1,^3^. The spike in the number of employees on short-time work in March and April 2020 is unprecedented and dwarfs even the strong increase following the Great Recession of 2007. Overall, about 1.9 million Swiss workers were registered for short-time work by the end of April 2020. This corresponds to more than every third worker in Switzerland being put on short-time work. Compared to this, the increase in unemployment had been much smaller with an increase of about 46,000 workers by the end of April 2020 compared to the year before. This increase, however, still corresponds to a steep surge of the unemployment rate by 43%.

In this project, we aim to shed light on the heterogeneous impact of the coronavirus lockdown on the Swiss labor market. For this, we have developed a metric named *lockdown index*, which measures a group’s initial exposure to the coronavirus restrictions. More specifically, we calculate the extent to which workers are restricted by the coronavirus by combining information on each occupation’s need for physical proximity to other people with rich, representative data of about 70,000 Swiss residents. For example, a choreographer (i.e., the occupation with the highest need for physical proximity in the data) is coded to be restricted by the coronavirus, whereas a fine artist (among the lowest need for physical proximity) is coded to be unrestricted by it. On top of this occupation-based assignment, we account for essential industries that were exempt from the government’s measures by coding workers in these industries as unrestricted. This results in a lockdown index, which can range between 0 and 1, and indicates the share of people in a certain group (e.g., canton, industry or income class) that is potentially restricted by the coronavirus lockdown^4^.

In the first part of the paper, we present heterogeneity of this lockdown index along several dimensions. This analysis suggests large differences in the impact of the lockdown across regions, industries, and income groups. Our lockdown index ranges from 39% of workers being restricted in Obwalden to 27% in Jura and from 60% in the hospitality industry to 14% in agriculture and by construction to 0% in public services. Moreover, we find that low- and middle-income workers (32 to 36%) are considerably more restricted than high-income workers (18%). In the second part of the paper, we test the explanatory power of the lockdown index for increases in short-time work and unemployment insurance claims by canton and industry until the end of April 2020. We find strong, positive correlations between the lockdown index and almost all of these short-term labor market outcomes and that the lockdown index alone can explain up to 20% of their cantonal variation and 58% of their industry variation.

There is a fast expanding literature analyzing the economic impact of the coronavirus. Similar attempts were made to explain the heterogeneous effect of the coronavirus based on the distribution of occupational characteristics. Dingel and Neiman (2020) used a home-office index to study the effect for the USA. This approach was then applied to other countries (e.g., Alipour et al. (2020) for Germany, Redmond and McGuinness (2020) for Ireland). Instead, our lockdown index relies on an occupation’s need for physical proximity instead of the ability to perform the job from home, which we find more suitable, at least in the Swiss setting^5^. Similar lockdown indexes were applied by Béland et al. (2020) and Mongey et al. (2020) to the USA, by Pouliakas and Branka (2020) to the EU, and by Alstadsæter et al. (2020) to Norway. Using individual-level administrative data for Norway, Alstadsæter et al. (2020) could report individual labor market outcomes (unemployment, short-time work) and link them to the occupational characteristics. Direct measures for labor market outcomes were also reported by Cajner et al. (2020) using administrative payroll data for the USA and Bartik et al. (2020) using survey data from small US businesses. Further empirical studies consider the impact of the coronavirus on other outcomes in Switzerland. Brülhart and Lalive (2020) study the effects on psychological and social suffering, Brülhart et al. (2020) the reaction of the self-employed, and Lalive et al. (2020) the behavior of job searchers.

This research originated in a real-time policy project that we started at the end of March 2020 shortly after the beginning of the lockdown in Switzerland. We reported the construction of our lockdown index and first results on April 17 in a blog post and on our project website^6^. We subsequently updated the results continuously as new information about unemployment and short-time work became available.

The remainder of this paper is structured as follows: Section 2 describes the methodology and data. Section 3 presents the results. Section 4 concludes.

## Methodology and data

### Lockdown index

To respond to the spread of the COVID-19 pandemic, policymakers implemented a lockdown policy aimed to enforce physical distances between people. These policies also heavily affected the work sphere. In Switzerland, many activities had to close or to severely limit their capacities during the peak of the pandemic. However, some jobs were more affected than others, partly because they inherently rely on physical proximity to other people to perform the necessary tasks. Moreover, some essential industries were exempt from the lockdown policy. We capture these differences in a metric we name lockdown index.

*Physical proximity requirements.* We base our index on the question about physical proximity requirements contained in the “work context” section of the Occupational Information Network (O*NET) survey. Table 1 provides an overview of the scores associated with different answers, along with our assignment of scores to the lockdown index.

**Table 1:** Assignment of physical proximity requirements to lockdown index

Question: To what extent does this job require the worker to perform		
job tasks in close physical proximity to other people?		
Answer	Score	Lockdown index (from this to next category’s score)
I do not work near other people (beyond 100 ft.)	0	0
I work with others but not closely (e.g., private office)	25	0
Slightly close (e.g., shared office)	50	0.5
Moderately close (at arm’s length)	75	1
Very close (near touching)	100	c1

This table lists the scores associated with different answers to the question about physical proximity requirements from O*NET (https://www.onetonline.org), along with our assignment of values for the lockdown index

The data contains the average answer score for 967 different occupations classified using the 6-digit American Standard Occupational Classification System 2010 (SOC-10). We assign a lockdown index value of 0 to average scores below 50, a value of 0.5 to scores equal or higher than 50 but lower than 75, and a value of 1 to scores equal to or higher than 75. To prepare the data for the crosswalk to the industry classification used in the Swiss labor market data, we then slightly reduce the granularity of the occupation classification to the 5-digit level SOC-10, taking an unweighted average of the 6-digit indexes and obtaining an index for 773 slightly broader SOC-10 groups.

*Crosswalk to the Swiss context.* We translate the index from the SOC-10 classification to the International Standard Classification of Occupations 2008 (ISCO-08), in order to use it in the Swiss context. We apply the crosswalk to the 4-digit ISCO-08 prepared by Hardy et al. (2018), taking an unweighted average in cases where many SOC-10 occupations compose an ISCO-08 occupation. We further calculate the index values also for higher ISCO-08 digits, computing the unweighted average of the corresponding subgroup. For example, the value of the 3-digit ISCO-08 category *Mining, Manufacturing and Construction Supervisors* (312) is composed by the equally weighted indexes’ values of the 4-digit occupations *Mining Supervisors* (3121), *Manufacturing Supervisors* (3122), and *Construction Supervisors* (3123). In turn, these occupations refer to the SOC-10 occupations *First-Line Supervisors of Construction Trades and Extraction Workers* (47-1011) and *First-Line Supervisors of Production and Operating Workers* (51-1011). We obtain the lockdown index for 9 1-digit, 39 2-digit, 105 3-digit, and 419 4-digit ISCO-08 groups.

*Essential sectors correction.* With the declaration of the “extraordinary situation” by the Federal Council on March 16, stringent measures were introduced that prohibited work at the usual workplace^7^. However, some essential sectors were excluded from these measures and remained more or less unconstrained despite their physical proximity character. We identify these sectors in our sample through the General Classification of Economic Activities 2008 (NOGA-08) and set a lockdown index of 0 for workers employed in these sectors. Although officially exempt from the government’s measures, we did not exclude hotels as they could in practice barely function^8^. The excluded sectors and the respective NOGA-08 groups are as follows: food stores, takeaway businesses, company canteens, and food home delivery services (4631-4639); pharmacies (4773); petrol stations (4730); banks (6419); post offices (5310–5320); public administrations and social institutions (8411-8430); railway stations and means of transport (4520, 4540); and hospitals, clinics, and medical practices (8610-8899).

*Public sector correction.* We also calculate an adjusted version of the lockdown index for our analysis of short-time work. Short-time work is aimed at avoiding sharp job losses by allowing firms to cut down labor costs during a crisis without firing employees. Firms are only eligible for short-time work if they can proof that the job would otherwise be at risk. Public sector jobs do no bear such a risk—with very few exceptions—and are hence generally no eligible for short-time work^9^. We therefore classify all public sector employees as unrestricted in the adjusted version of the lockdown index.

### Home-office index

In an innovative attempt to assess the heterogeneous impact of the coronavirus lockdown on the US workforce, Dingel and Neiman (2020) used a similar method and calculated the shares of workers that can work from home along several characteristics (e.g., metropolitan areas or socio-demographic characteristics). Their calculation is based on a number of questions from the O*NET database, including (but not limited to) whether an occupation requires daily “work outdoors” or that “operating vehicles, mechanized devices, or equipment” is very important to that occupation’s performance. If so, such occupations are classified as not being suitable for home-office. The resulting metric may be referred to as a “home-office index,” ranging between 0 and 1, with 1 denoting that all workers of that group can work from home.

There are two main differences between our lockdown index and this home-office index: first, our index relies solely on an occupations need for physical proximity to other people as opposed to the possibility to perform tasks at home. This difference is crucial for several large occupations. On the one hand, truck drivers, food delivery drivers, many agricultural workers, and some occupations in the construction sector can impossibly work from home, yet remained largely unrestricted by the lockdown policy measures. On the other hand, other professions such as music teachers may be able to work from home, but were nevertheless restricted due to the close proximity that is necessary to perform this job. Table 2 presents several examples of occupations for which the lockdown index and the home-office index are either in line or in contradiction with one another. Second, as mentioned above, we correct the lockdown index for essential sectors that were explicitly exempt from the lockdown measures. These include several occupations that cannot be performed from home, such as doctors, nurses, or cashiers in food stores, but that were unrestricted by the lockdown measures.

**Table 2:** Lockdown vs. home-office index

ISCO code	Occupation title	Lockdown index	Home-office index	Type
2512	Software developer	0.0	1.0	Both unrestricted
2611	Lawyer	0.0	1.0	Both unrestricted
2631	Economist	0.0	1.0	Both unrestricted
2622	Librarians	0.4	0.5	Both partially restricted
2642	Journalist	0.5	0.5	Both partially restricted
4110	Office clerks	0.4	0.5	Both partially restricted
2250	Veterinarians	1.0	0.0	Both restricted
5132	Bartender	1.0	0.0	Both restricted
7112	Bricklayer	1.0	0.0	Both restricted
2145	Chemical engineer	0.0	0.0	Contradiction*
6130	Farmer	0.0	0.1	Contradiction*
9112	Office and hotels cleaner	0.1	0.0	Contradiction*
2354	Music teacher	1.0	1.0	Contradiction**

^*^Occupations restricted for the home-office index, but unrestricted for the lockdown index

^**^Occupations unrestricted for the home-office index, but restricted for the lockdown index

Although we view the home-office index to be less suitable for assessing the heterogeneous impact of the lockdown on the Swiss labor market, we report results for this alternative index in the appendix^10^.

### Data

*Swiss Labor Force Survey 2018 (SLFS).* We use data from the SLFS for the year 2018 to estimate the characteristics of the lockdown index on the Swiss labor market. The SLFS is an individual survey collected by the Swiss Federal Statistical Office (BFS), which is representative of the Swiss permanent resident adult population^11^. We focus on the employed population, thus excluding unemployed respondents, people in education and retirees from the sample. Moreover, we exclude respondents with a missing occupation classification. When calculating averages for different subgroups, we apply sampling weights converted to full-time equivalents.

Occupational Information Network (O*NET). We use data on occupational task requirements from O*NET, a database containing detailed descriptions of American occupations. In particular, we focus on a question about the need for physical proximity from the section “work context” (see Table 1 for more information).

*Structural Business Statistics (STATENT).* To account for the size of sectors and regions we base on the information collected by the BFS in STATENT. This administrative data covers all employees and self-employed people contributing to social security (old-age and survivors’ insurance, AHV), thus all workers with an annual income of more than 2300 Swiss francs.

*State Secretariat of Economic Affairs (SECO) labor market reports.* To test the explanatory power of the lockdown measures for short-term labor market outcomes in Switzerland, we use information about the unemployment rate and about the number of applications for short-time work by canton and industry. Data on unemployment is released by the SECO on a monthly basis. SECO considers as unemployed every job seeker who is registered at a regional job placement office. For short-time work, we use data on approved pre-registrations for short-time work compensation that were submitted to the cantonal administrative offices between March 1 and April 28, 2020. Normally, SECO reports detailed data on paid-out short-time work compensation only with a 3-month time lag. Due to the urgency of the current situation, SECO made these preliminary data on approved pre-registrations available at the end of April 2020.

The actual number and geographical distribution of paid-out compensations may substantially differ from those for several reasons: first, employers with several establishments are supposed to pre-register with each canton where establishments are located. However, employers who operate countrywide often pre-register only at the headquarter’s canton. For this reason, cantons housing headquarters of large companies that operate throughout several cantons (e.g., Basel-Stadt) may receive more pre-registrations than will eventually be paid out there. In fact, Fig. 25 shows that for some canton-industry combinations, the number of short-time work registrations exceed the number of actually employed workers many times over. It is even possible that some of these large companies, in error, file requests both at the headquarters and the individual establishments. Such potential double counts will only be corrected once compensations will be paid out. Second, up until April 28, 2020, almost all pre-registrations had been approved in an urgent procedure. It is possible that a considerable share of these pre-registration may eventually either not be claimed or paid out for other reasons. These reported numbers may therefore be interpreted as an upper bound of the short-time work compensations that will eventually be paid out. Third, the leniency in the final approval of short-time work compensation varied substantially in the past (up to 40 percentage points during the Great Recession of 2007). The geographic distribution of the eventually paid short-time work compensations may therefore substantially differ from the one reported here. This figure, however, will only be known after a period of three months when the firms claim the benefits and provide supporting documentation.

*Comparis.ch internet user data.* In cooperation with *comparis.ch*, the most popular consumer-empowerment websites in Switzerland, we analyzed user data from their so-called “short-time work calculator” (Kurzarbeitsrechner in German) launched at the end of March. This tool that lets users enter their current salary and other information and calculates the implied salary they would get when being put on short-time work. Every visit to this website can be attributed to a single user and, via the Internet Protocol (IP) address, to a canton. About 90,000 people made use of this service by April 20, 2020.

In our analysis, we show the share of short-time work calculator users of total comparis.ch users in that canton. This way, we account for cantons’ different population sizes and different popularity of comparis.ch. We only report values for the German speaking cantons, as the short-time work calculator was only actively promoted in the German speaking part of Switzerland (via “20 Minuten”, one of the news portals with the highest coverage in Switzerland).

## Results

In this section, we calculate the lockdown index for Switzerland and link it to the observed development on the labor market during the COVID-19 pandemic. In Section 3.1, we report differences in the lockdown index by industry, region, and socio-demographic characteristics. In Section 3.2, we show that the lockdown index is highly correlated with observed changes in unemployment and short-time work. The lockdown index therefore provides insight into the structural reasons for the observed changes during the crisis. For example, observed regional differences in unemployment changes can be largely explained by regional differences in the industrial structure. Moreover, the index sheds light on the ability of industries, cantons or other groups to operate at full capacity when hygiene measures related to physical proximity will still be in place after strict lockdown measures are relaxed. Finally, the lockdown index teaches us about the differential impact of the lockdown on different groups of society for which administrative data is either unavailable or becomes available only several months later^12^.

### Heterogeneity of lockdown index

In this subsection, we report differences in the lockdown index by industry, region, and socio-demographic characteristics.

**Fig. 2:**
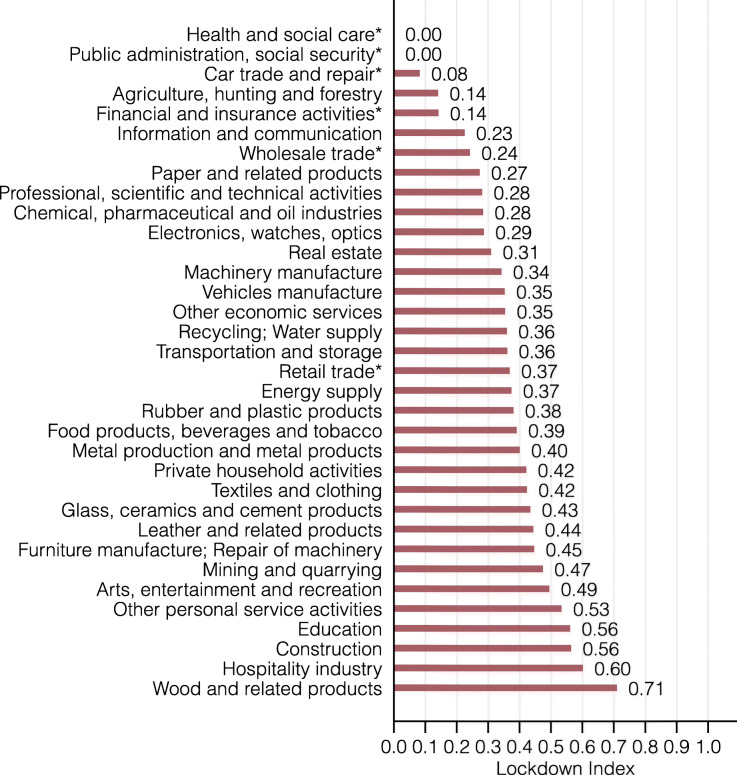
Lockdown index by industry

*Industry-level differences.* As a first step, we present the lockdown index for 34 industries in Fig. 2. There is striking heterogeneity in the lockdown index across industries. Among the larger industries, the hospitality, construction, and education industry are most affected by the lockdown, with more than 56% of workers being restricted by the coronavirus lockdown^13^. In contrast, agriculture, financial services, and the information and communication industry are relatively unaffected, with less than 23% of workers having to work close to other people. By construction, the essential sectors health and social care and public administration are unrestricted.

Such differences in the lockdown index arise from different compositions of occupations across different industries. For example, the hospitality industry relies heavily on waitresses/waiters, bartenders, or cooks, all of whom require close contact with customers or co-workers to perform their job properly. In contrast, the financial services industry employs many managers, data processing clerks, or secretaries, all of whom work largely with computers, without the explicit need to be physically close to others.

**Fig. 3:**
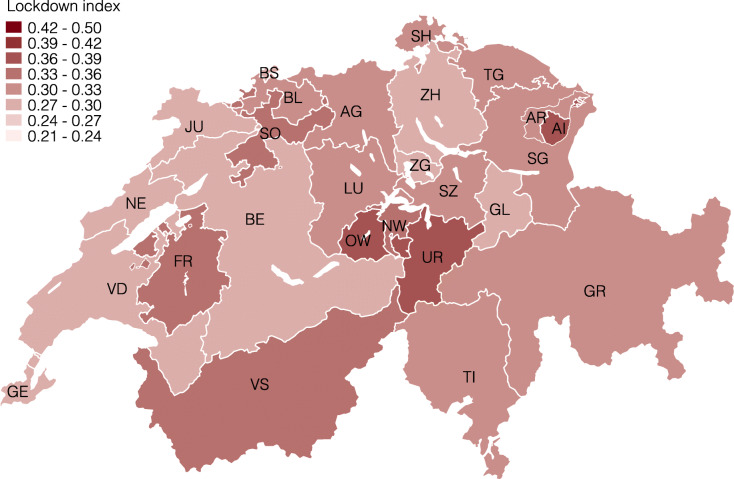
Lockdown index by canton

*Regional differences.* Next, we present the lockdown index at several levels of geographic aggregation. We start by showing the index for the 26 cantons in Fig. 3 and find considerable regional heterogeneity. For example, the cantons Obwalden (0.39), Appenzell Innerrhoden (0.38), and Uri (0.37) are the most restricted by the coronavirus lockdown, whereas Jura (0.27), Zug (0.28), and Geneve (0.28) are the least restricted, followed by Zürich (0.29) and Basel-Stadt (0.29).

These differences reflect the specialization of different cantons in specific industries, which are, in turn, differentially restricted by the lockdown. For example, the cantons with the highest values are all intensive in the hospitality and tourism or construction industry, which are highly affected, and less intensive in health and social care or public administration, which feature a low lockdown index. The opposite is true for cantons with low lockdown indexes, with Jura being highly specialized in the watch industry, Zürich in financial services, or Basel-Stadt in pharmaceuticals and chemicals.

Cantonal borders are, however, not ideal to assess the impact of shocks on local labor markets, as political boundaries have often no economic relevance. One reason for this are substantial commuting ties between cantons such for example between Basel-Stadt and Basel-Landschaft. In this example, it is more relevant to understand the change in labor demand in the combined labor market of both cantons. We therefore use two official classifications of local labor markets that take into account such commuting ties by clustering municipalities with strong commuting ties within and weak commuting ties across. The broader one clusters municipalities into 16 large labor market regions (Arbeitsmarktgrossregionen in German). The finer one clusters municipalities into 101 labor market regions (Arbeitsmarktregionen).

**Fig. 4:**
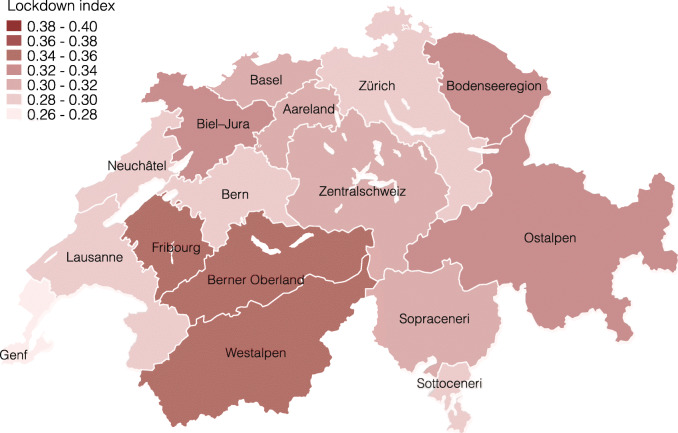
Lockdown index by large labor market region

**Fig. 5:**
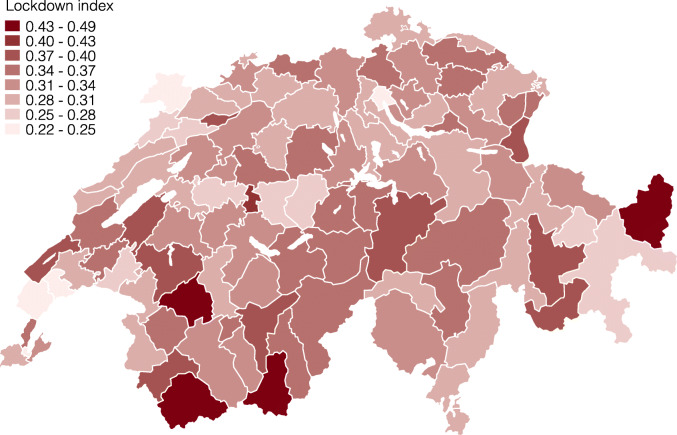
Lockdown index by labor market region

Figures 4 and 5 present the lockdown index for both of these local labor market definitions^14^. As we expected, the differences become smaller when taking averages of fewer, larger units in Fig. 4, and vice versa in Fig. 5. At the large labor market region level, the regions around Zürich and Geneve remain among the least restricted, whereas the regions Westalpen, Berner Oberland, and Fribourg are the most restricted. The differences at this level are still considerable, ranging from 27 to 35% of workers being restricted. Finally, Fig. 5 repeats this exercise at the smaller, labor market region level. At this level, the lockdown index ranges between 22 and 49% of workers being restricted.

**Fig. 6:**
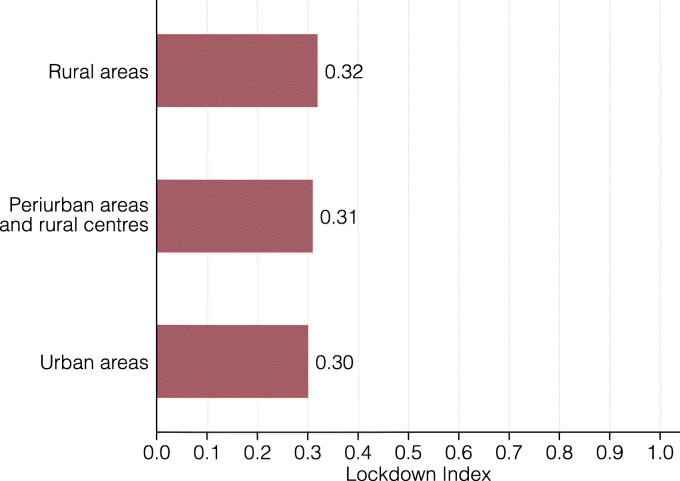
Lockdown index by population density

The level of aggregation in Fig. 5 also gives a first impression of a potential urban-rural gap in terms of the lockdown index. Visual inspection does not strongly support any such differences. Nevertheless, we explore this more directly in Fig. 6, where we calculate the lockdown index for the set of municipalities that are officially classified as urban, rural, or in between. Also in this breakdown, there is no strong difference in how urban and rural areas are restricted by the coronavirus lockdown.

*Socio-demographic differences.* To assess the heterogeneous impact of the coronavirus lockdown on the Swiss labor force, we explore the heterogeneity of the lockdown index by income group, age, gender, and civil status.

**Fig. 7:**
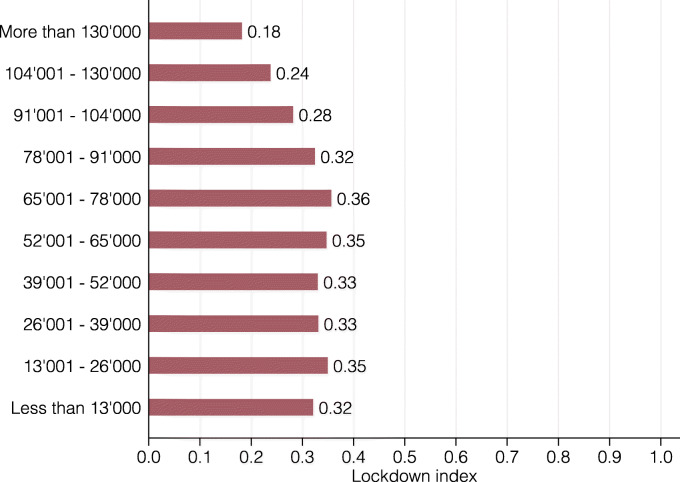
Lockdown index by income group

Figure 7 presents the lockdown index for ten income groups. Income is defined as annual gross income of workers without a side job. Again, there is substantial heterogeneity, with the share of workers that are restricted by the lockdown ranging from 18 to 36%. In particular, low- and middle-income individuals (less than CHF 91,000) are strongly affected with values ranging from 32 to 36%. In contrast, high-income individuals (more than CHF 91,000) are the least affected, with the lowest index for the highest income group. This is plausible, given that many low-income service occupations such as waitresses/waiters or construction workers rely on physical proximity to do their job and that high-income jobs such as managers, software developers, or lawyers entail mostly abstract tasks that may be performed with close proximity to other people.

**Fig. 8:**
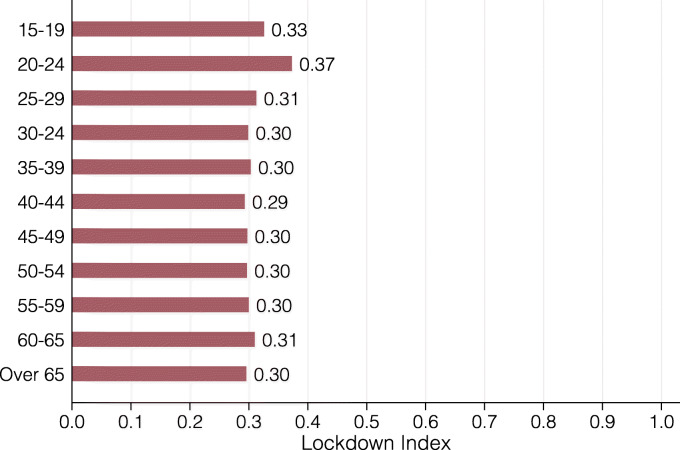
Lockdown index by age group

**Fig. 9:**
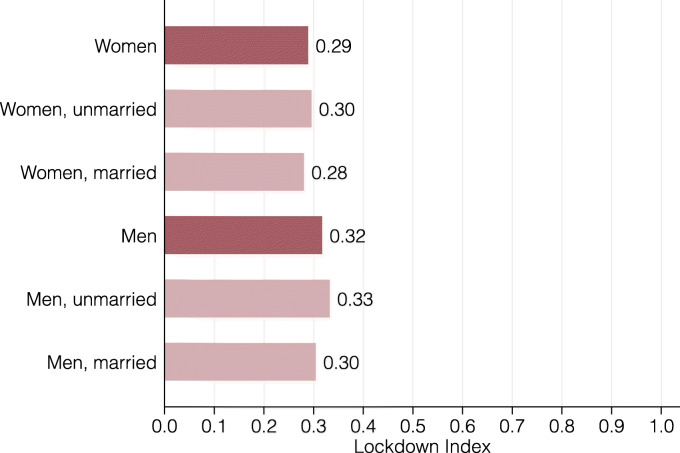
Lockdown index by gender and civil status

In Fig. 8, we present the lockdown index for eleven age groups. The different age groups have astonishingly similar index values with the exception of 20-24 year olds, who are substantially more restricted by the lockdown. This is explained by this group’s especially high concentration in side jobs in restaurants, bars or the retail sector. Figure 9 presents the lockdown index by gender and civil status. On average, women are slightly less restricted by the coronavirus lockdown than men, though not to a large extent. For both genders, single people are somewhat more affected than married ones or those in a civil union. We conjecture that this is due to both the lower average age of single people and children in the household, which tend to incentivize people to sort into jobs that can be performed from home.

### Explanatory power for employment changes during lockdown

In this subsection, we study the explanatory power of the lockdown index for the short-run labor market outcomes during the lockdown measures.

*The Swiss labor market.* Historically, the unemployment rate in Switzerland has been relatively low, ranging between 1.7 and 3.9% since 2000, with an average of 3.0%. Compared to other European countries, Switzerland has a highly flexible labor market with comparatively short notice periods for the cancelation of work contracts. In general, firms have two main margins of adjustment when they want to or have to reduce labor costs. First, they may lay off workers, who will then, after the notice period has elapsed and if they were unable to find a new job in the meantime, file for unemployment insurance at the regional employment center. Notice periods vary by tenure, with 1 month for workers in their first year of employment, 2 months for those in the second to ninth year, and 3 months for workers with higher tenure at this employer. Unemployment appears in our data after employment is actually terminated.

Second, employers may file for short-time work compensation of certain employees if they face temporary drops in production due to external factors. If on short-time work, the employee remains employed, but at a reduced workload, which may be as low as 0%. As compensation for the reduction in workload, the employee receives a subsidy of 80% of her foregone salary. Short-time work compensation is paid out during a maximum of 12 months over a 2-year period. Short-time work requests pass three stages: employers have to *pre-register* for short-time work compensation, pre-registration then gets *approved* or rejected, and finally, short-time work compensation is *paid out* to employers^15^.

*Explaining the rise in short-time work.* In response to the coronavirus crisis, the number of workers on short-time work compensation has soared to unprecedented heights. As of April 28, 2020, 1.9 million registrations had been approved, i.e., more than every third individual in the Swiss labor force.

**Fig. 10:**
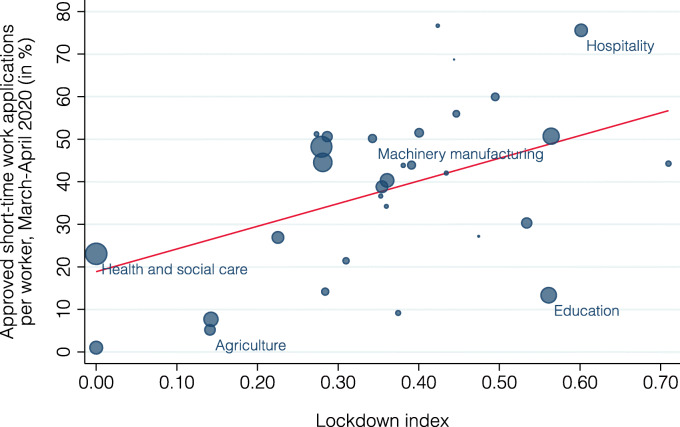
Relationship between lockdown index and short-time work by industry

We examine next how well the lockdown index is able to explain the industry-level differences in the prevalence of short-time work registrations. Figure 10 plots the relationship between the share of workers on short-time work and the lockdown index; bubble size indicates an industry’s share of national employment. The red line plots the fitted values from a regression of the share of short-time workers on the lockdown index and a constant, weighted by an industry’s share of national employment. There is a strong positive correlation (slope = 53.3, standard error = 22.7, *p* value = 0.03) with the lockdown index explaining 30% of the variation in short-time work^16^. There are, however, some notable deviations such as the education industry that is predicted to have a much larger share of short-time workers than observed.

**Fig. 11:**
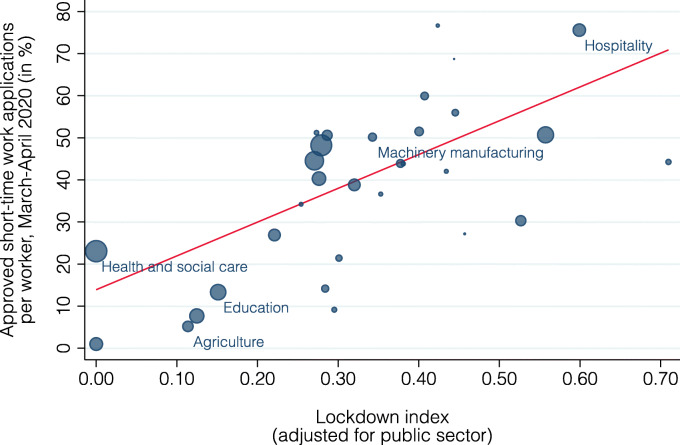
Relationship between adjusted lockdown index and short-time work by industry

This discrepancy may be due to the fact that many jobs in education are in the public sector. According to the guidelines of the SECO, short-time work compensation is only allowed if a job would have otherwise disappeared, which is not the case for most public employers. To account for this specificity, we adjust the lockdown index by classifying all workers in the public sector as unrestricted. Figure 11 presents the relationship between this adjusted lockdown index and the share of workers in short-time work (slope = 80.3, standard error = 17.1, *p* value = 0.00). This adjustment improves the explanatory power of the lockdown index considerably to 58%.

**Fig. 12:**
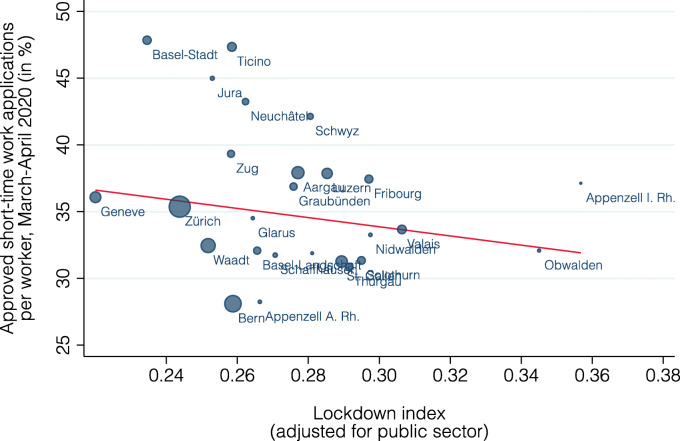
Relationship between lockdown index and short-time work by canton

Figure 12 shows how the lockdown index is related to the observed cantonal variation in short-time work. If anything, there is a negative relationship between the two variables (slope = − 34.4, standard error = 30.7, *p* value = 0.28, *R*^2^ = 0.02).

Why is the lockdown index able to explain the increase in short-time work so well by industry but not by canton? First, there are systematic reporting errors regarding the canton in which short-time work is registered as explained in the data section 2.3. Second, the coronavirus is more prevalent in Swiss cantons (Basel-Stadt, Jura, Geneva, and Ticino) sharing long borders with neighboring France and Italy, two of the most affected countries in Europe. The experience from close neighbors has likely caused stricter enforcement of the lockdown and a stronger response of firms. Third, the SLFS data does not include cross-border commuters. If commuters substantially differ from residents in their distribution across occupations, the lockdown index may not perfectly capture the exposure for border cantons with many cross-border commuters.

**Fig. 13:**
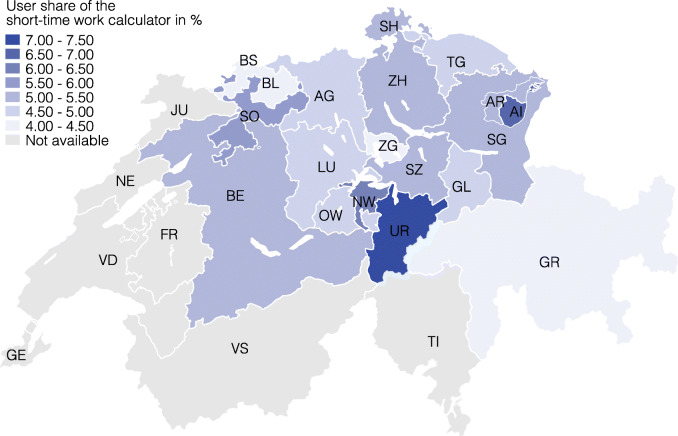
Share of comparis.ch users using short-time work calculator by canton

Partly because of these limitations, we conduct an alternative analysis to assess the regional distribution of short-time work. We use the number of visits on a web-based short-time work salary calculator from comparis.ch (see the data section 2.3). The use of salary calculator differs substantially across cantons (see Fig. 13). The cantons Graubünden, Basel-Stadt, Basel-Landschaft, and Zug had the lowest usage with shares of less than 4.5%. The canton Uri features the highest value with more than 7%. Moreover, Appenzell Innerrhoden and Nidwalden have high shares with more than 6%.

**Fig. 14:**
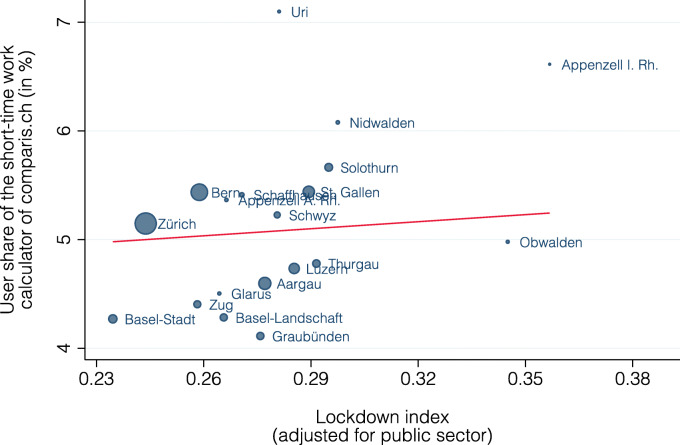
Relationship between lockdown index and comparis.ch user share using short-time work calculator by canton

Figure 14 shows the relationship between the lockdown index and the share of comparis.ch users that use the short-time work calculator at the cantonal level. There is a slight, but insignificant positive correlation (slope = 2.2, standard error = 5.0, *p* value = 0.67), with the lockdown index alone being able to explain about 9% of the variation in the use of the short-time work calculator.

*Explaining the rise in unemployment.* By the end of April 2020, the number of registered unemployed individuals has risen by 46,115 compared to the year before. Despite this historically large increase in unemployment, this remains is a relatively small response compared to the additional 1.9 million individuals in short-time work. Reasons for this may be that firms rely more on short-time work instead of laying off workers to save labor costs, but also that laid-off workers may still be employed due the notice periods of several months for the cancelation of work contracts. Because of the latter, it is likely that a large part of the increase in unemployment in these first 2 months after the start of the coronavirus crisis is attributable to fewer job openings rather than more layoffs.

**Fig. 15:**
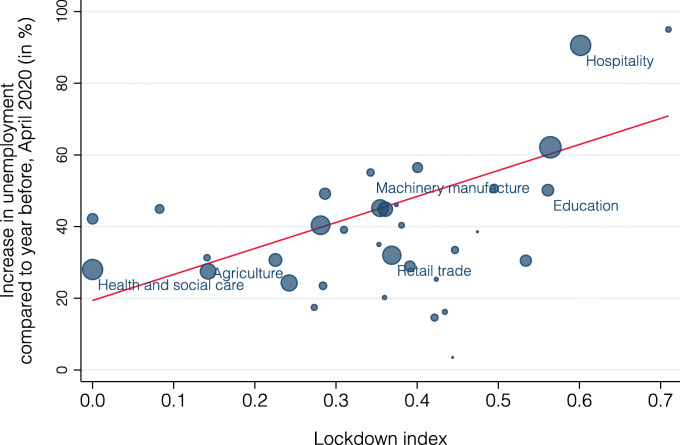
Relationship between lockdown index and unemployment by industry

**Fig. 16:**
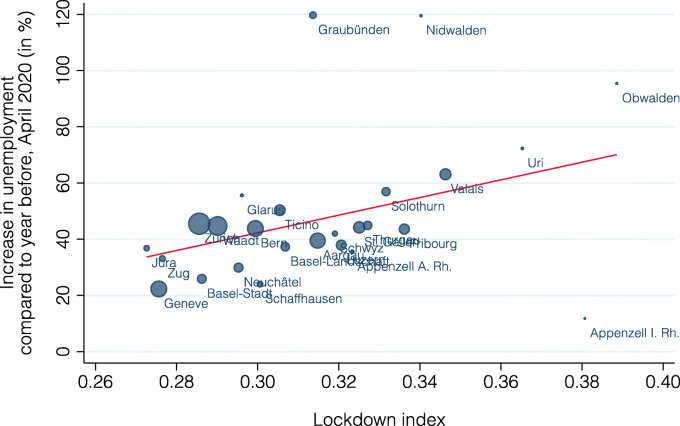
Relationship between lockdown index and unemployment by canton

Figure 15 presents the relationship between the lockdown index and the percentage increase in unemployment by the end of April 2020 (compared to April 2019); bubble size now indicates each industry’s share of national unemployment. There is again a clear positive correlation (slope = 72.5, standard error = 23.3, *p* value = 0.00). The lockdown index explains about 47% of the variation in unemployment changes across these 34 industries. Figure 16 repeats the same analysis at the cantonal level. There is a clearly visible positive correlation also at this level (slope = 314.1, standard error = 119.8, *p* value = 0.02), with the lockdown index being able to explain 20% of the variation. It has the lowest explanatory power for the cantons Nidwalden, Appenzell Innerrhoden, and Graubünden. Similarly to our analysis on short-time work above, the lockdown index is better able to explain differences across industries than across cantons.

**Fig. 17:**
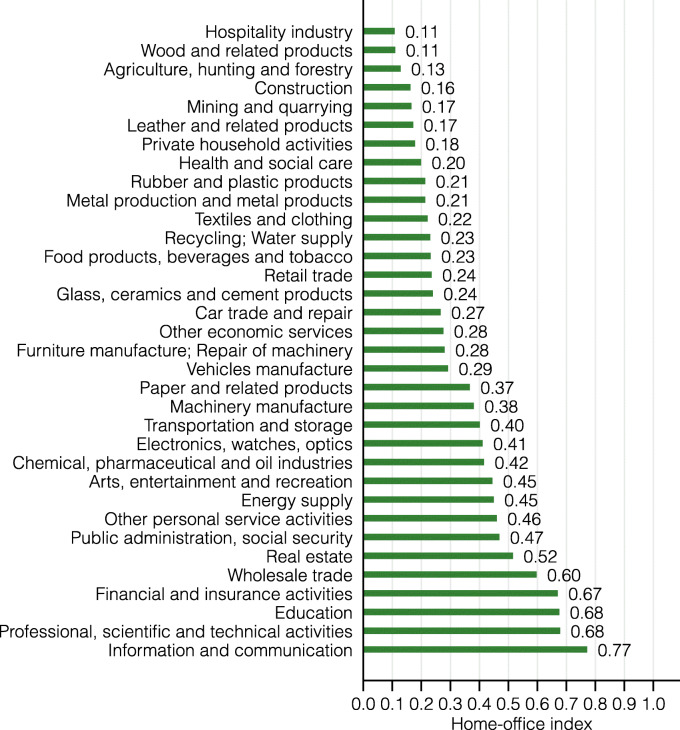
Home-office index by industry

## Conclusion

In this project, we assess the heterogeneous effects of the coronavirus lockdown on the Swiss labor force. For this, we construct a lockdown index, which measures whether workers perform jobs that require physical proximity to other people and whether they are employed in essential sectors that were exempt from the policy measures. The lockdown index suggests that the impact of the lockdown is highly heterogeneous across cantons and industries, as well as different socio-demographic groups. The lockdown index also serves as a proxy for short-term labor market outcomes. For example, it explains a significant part of the increase in short-time work compensation or unemployment at the industry and cantonal level. One exception is the increase in short-time work at the cantonal level, which differs substantially from the one predicted by the cantonal job composition using the lockdown index. As the observed short-time work data is yet provisional, we will have to reconsider this question when the final data will be published in a few months. Given that jobs with high physical proximity requirements will find it more difficult to abide by the increased hygiene measures after the actual lockdown, the lockdown index may also be informative about the heterogeneous impact of the coronavirus in the medium term.

We view the lockdown index also as a suitable tool to inform political decisions on measures targeted at mitigating the distributional distortions caused by the coronavirus. In particular, our analysis shows which socio-demographic groups are most heavily affected by the coronavirus and the corresponding lockdown policies. We find that higher income groups are less affected, that age groups are similarly affected except for 20–24 years old who are substantially more affected, and that there only small differences between men and women and between urban and rural regions.

We consider this analysis only a first step to shed light on the question which regions, industries or groups of society are most heavily affected by the coronavirus and the policy measures that came along with it. In the long run, the extent of the effect depends on several factors that are not considered in this project. First, it will be crucial which groups are going to be supported by the government. Second, both the supply and demand side is not only affected by policy measures in Switzerland, but also by the situation in other countries via global value chains. Third, the extent to which industries will be able to regain some of the demand that was lost during the crisis will differ depending on the types of products. For example, it is conceivable that durable industries such as furniture, automotive, or electronics may be able to gain back some of the lost demand during the recovery and that this will be considerably more difficult for producers of non-durables such as restaurants or food and beverage producers.

**Fig. 18:**
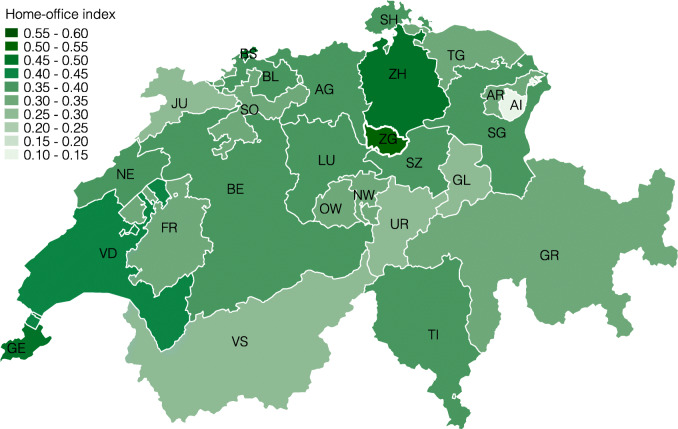
Home-office index by canton

**Fig. 19:**
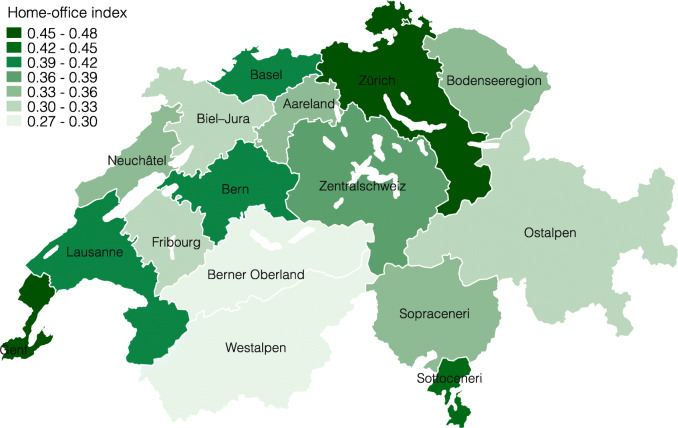
Home-office index by large labor market region

**Fig. 20:**
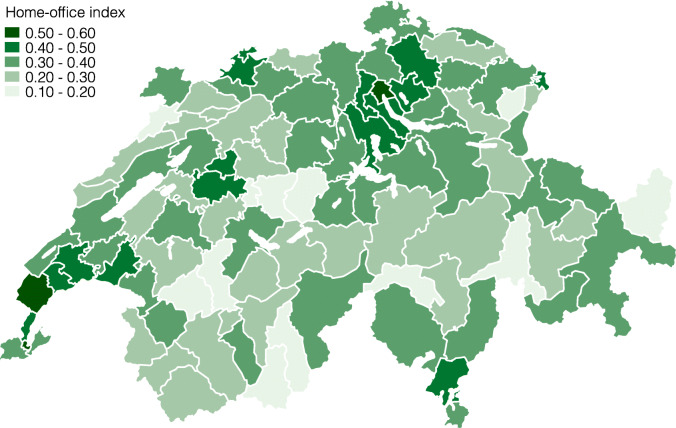
Home-office index by labor market region

**Fig. 21:**
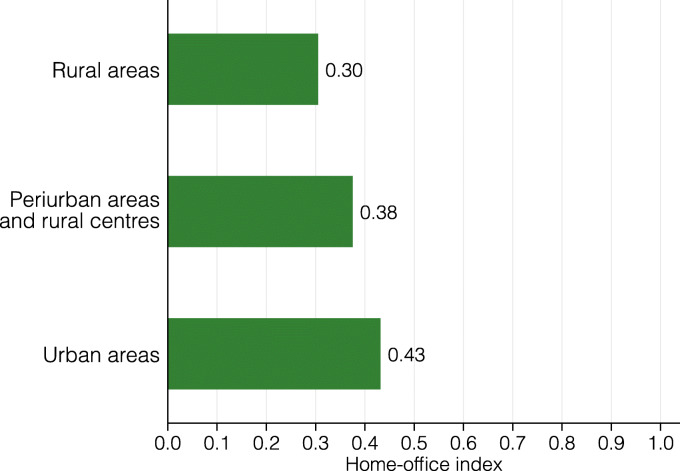
Home-office index by population density

**Fig. 22:**
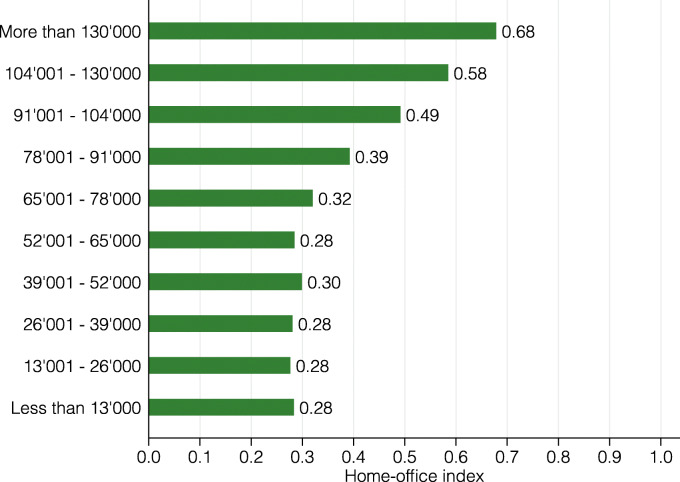
Home-office index by income group

**Fig. 23:**
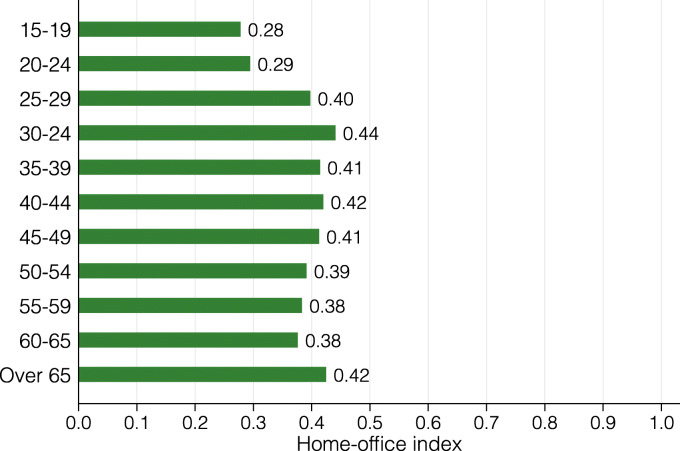
Home-office index by age group

**Fig. 24:**
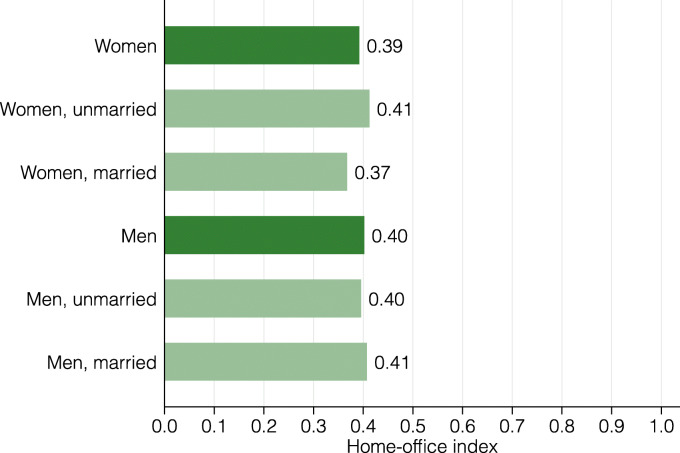
Home-office index by gender and civil status

**Fig. 25:**
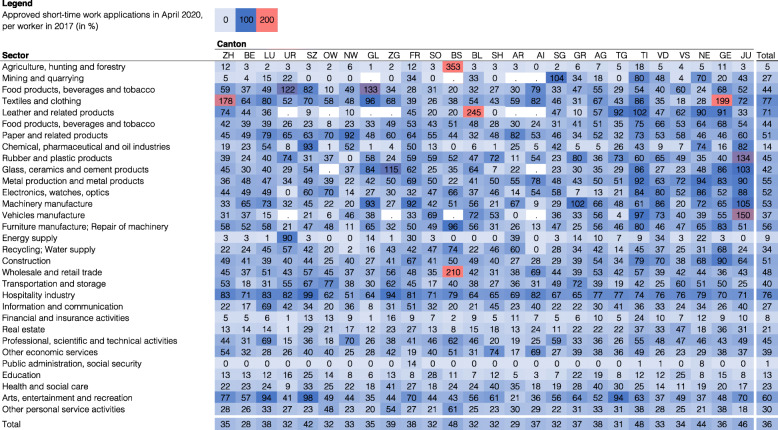
Approved pre-registrations for short-time work compensation as a share of all workers in 2017 by canton and industry

Although access to individual-level administrative data in Switzerland has been significantly eased during the last decade, this real-time policy project also showed us that access is still limited and slow compared to other European countries. We worked with individual data from a pre-crisis survey and highly aggregated official unemployment and short-time work figures. At the same time, researchers in Norway (Alstadsæter et al. 2020) were able to observe the labor market status of the universe of Norwegian workers in administrative data along with detailed information about worker, household, job, and firm characteristics. They were even able to match information about income payments and bank account balances. This allowed them to study the real heterogeneous effects of the coronavirus already during the crisis. Such fast and detailed access to available administrative data—while protecting privacy in a highly secured environment—allows for more robust evidence-based policies even in uncharted territory such as the current coronavirus crisis.

## Appendix

## Data Availability

The data used in this study are confidential data from the BFS, SECO, and comparis.ch. Upon publication, all code and generated data as well as a description on how to obtain the confidential raw data can be downloaded on a publicly available repository on Harvard Dataverse (10.7910/DVN/YZ2BKS).

## Abbreviations

BFS: Swiss federal statistical office
COVID-19: Coronavirus disease 2019
ISCO-08: International standard classification of occupations 2008
NOGA-08: General classification of economic activities 2008
O*NET: Occupational information network
SECO: Swiss state secretariat of economic affairs
SLFS: Swiss labor force survey 2018
SOC-10: American standard occupational classification system 2010
STATENT: Structural business statistics

## Footnotes

1 According to the European Centre for Disease Prevention and Control (ECDC), Switzerland had the second highest number of confirmed cases per capita of all major European countries on March 17, 2020.

2 See also Eichenauer and Sturm (forthcoming) for more details on the content and timing of the measures taken by the Swiss government.

3 Short-time work describes a reduction or complete stoppage of the contractual workload for up to 12 months. If in short-time work, the employee continues to receive the proportional salary for the reduced workload, plus 80% of her foregone salary as compensation. This compensation is financed by the unemployment insurance fund, but paid out by the employer.

4 The main objective of the lockdown index is to estimate the effects of the Swiss lockdown policy. However, it possibly also approximates the heterogeneous economic effects of the coronavirus more broadly, as people may have similarly avoided close proximity to others even in the absence of a lockdown policy.

5 The Swiss government’s policy was directly aimed at reducing physical contact between people, not necessarily at having them work from home. For example, delivery truck drivers or farmers were largely able to continue their work, despite them not being able to work from home. Based on the method used in Dingel and Neiman (2020), Rutzer and Niggli (2020) calculate the shares of people, who can work from home, along several dimensions for Switzerland.

6 http://www.batz.ch/2020/04/wo-der-lockdown-am-staerksten-zu-spueren-ist/and https://wwz.unibas.ch/de/appliedeconometrics/coronavirus/

7 https://www.admin.ch/gov/en/start/documentation/media-releases.msg-id-78454.html

8 Indeed, the Tourist Accommodation Statistics (HESTA) reported only 1,264,231 hotel accommodations in Switzerland for March 2020, a drop of 62.3% with respect to the 3,352,687 accommodations in the same month of the previous year. See https://www.bfs.admin.ch/bfs/en/home/statistics/tourism/tourist-accommodation/hotel-accommodation.html

9 Although the regulations and guidelines for short-time work generally rule out application by public sector enterprises like hospitals and public transport, there could have been many applications in the initial applications according to media reports. These approved applications will likely not be paid out eventually.

10 Moreover, we confirm that the lockdown index is indeed able to explain more of the actual industry- and canton-level variation in unemployment and short-time work increases. Results for these analyses are available upon request.

11 Note that the Swiss labor force contains about 330,000 cross-border commuters from neighboring countries, who are not included in this sample. The occupational structure of these may differ from Swiss residents. Therefore, the lockdown index calculated in this paper should be interpreted as the exposure of the Swiss resident population, as opposed to the Swiss labor force, to the coronavirus lockdown.

12 For example, official unemployment numbers are not reported by income bracket or civil status. Moreover, short-time work applications take several months to be processed, such that data on paid-out short-time work will only become available with a considerable time lag.

13 In the strict sense, the lockdown index does not exactly correspond to the share of workers that are restricted by the coronavirus lockdown, because some occupations feature values between zero and one. This is due to both our assignment of intermediate values for occupations with moderate proximity requirements and the crosswalk to a broader industry classification.

14 Results have to be interpreted with caution when based on fewer than 50 SLFS observations. This is the case in 14 out of 101 labor market regions, namely Bagnes, Crans-Montana, Faido, Leuk, Meiringen, Moutier, Saignelégier–Le Noirmont, Scuol, St. Moritz, Thusis, Val-de-Travers, Vaz/Obervaz, Zermatt and Zweisimmen–Lenk.

15 Cantons may deny requests, for example, if a firm’s problems are viewed to be rather structural than temporary. During the Great Recession of 2007, the approval rate of short-time work pre-registrations varied between 55 and 100% across cantons (Kopp and Siegenthaler 2019). In March and April 2020, this approval rate has been close to 100% in all cantons. Reasons for this may be the substantially higher volume of pre-registrations and the increased sense of urgency in this crisis. Cantons may plan to reconsider these approvals more thoroughly before eventually paying out compensations.

16 We report heteroskedasticity robust standard errors.
